# A Case of Inversion of the Uterus

**Published:** 1899-02

**Authors:** F. A. Schmitt

**Affiliations:** La Grange, Texas


For Texas Medical Journal.

### A Case of Inversion of the Uterus.

BY F. A. SCHMITT, M. D., LA GRANGE, TEXAS.

During a term of thirty-five years of active practice as a general practitioner, it has been my fate to encounter two obstetrical cases of the gravest nature, namely: two cases of inversion of the womb, one having been but a partial or introversion, the last case, however, having been a complete inversion of this organ.

The former happened during my early years as a practitioner, and I was then so fortunate as to have the assistance of an experienced obstetrician as far as diagnosis and the handling of the case was concerned. My last case I had only a few weeks ago, and was caused, as is usually the case, from pulling at the funis of an adherent placenta; in this case the sinner being an ignorant but aggressive and daring nurse.

As this case, aside from its formidableness and rareness, presents points of interest from a physiological as well as from a social point of view, I take the liberty of giving you the particulars of the case.

In the evening of January 1st, 1899, I was asked to prepare myself to attend to Mrs. A. M., a Syrian woman, mother of several children, who was likely to be confined during the following night, as the husband suggested. Consequently, at 3 a. m. of January 2nd I was called, and soon after was on my way to the dwelling house of the people, a distance of six blocks. Several of the family met us half way and urged us to make haste, alleging the woman to be in a dying condition, she having given birth to a child but a short while ago.

It was a cold morning, and on arrival, I was ushered into a small —perhaps 8 x 8—room, lacking fireplace, stove, or even bed, but a woman crouching in a rocking chair, surrounded or supported by three or four wildly gesticulating and loudly shrieking women, none of whom could speak sufficient English to give any information in regard to the case, the dim flame of a smoking kerosene lamp merely giving light enough to illuminate the dreadful condition of the woman, her offspring, and the primitive surroundings.

Immediately instituted examination revealed the following conditions: She had given birth to child which was alive and lying on the cold, bloody floor between the woman's feet and still attached to placenta, placental mass outside of vulva and yet adherent to womb which, on careful palpation of pubic region and digital examination, I pronounced inverted; the woman, from effect of shock, in an almost collapsed condition. I had little time to reflect, and I, more instinctively than anything else, and while the woman was still in the chair, cut the cord and severed the child, which was cold and cyanosed, but still alive, from its mother, and forcibly detached the placenta.

Meanwhile I had directed the people to furnish a mattress, upon which I placed the now bleeding woman. At this time a soft mass, similar to placental mass, appeared outside of vulva, which I, on reflection, determined to be prolapsed vagina. I then instituted measures for the reversion and reduction of the uterus. Accordingly I pushed prolapsed vagina back, and with my right hand cone shaped, placing my left hand above the pubes to support my efforts from below, I succeeded in replacing the womb to its natural site, and felt the organ contract around my hand, which I then slowly withdrew, *the relaxed condition of the woman's whole muscular system aiding me, undoubtedly, in bringing about this fortunate change of conditions, in comparatively but little time or exertion.*

The time occupied in performing the services can be contemplated by the fact that I returned home one hour and fifty minutes after leaving, spending most of this time in successful efforts of reviving the woman by administering hypodermics of whiskey, applying sinapisms, etc. I left her in as comfortable and promising condition as could have possibly been expected under the circumstances, she having regained consciousness and her pulse beating at the rate of 130 per minute at the wrist, which previously could not be perceived at all; womb having contracted nicely, contours of which now readily could be made out, and no hemorrhage from same.

To the husband, who could speak English tolerably well, I gave further instructions, and besides a rather gloomy prognosis, as the exposed internal parts had in the hurry been handled by myself not aseptically by any means, and besides had been exposed, before my arrival, to the touch with rough and dirty hands by the so-called midwife, who in her outlandish way told of her deeds, acts, and behavior in the premises .

On calling five hours later, I found the woman in another room, placed in bed, and seemingly comfortable, in the act of nursing her tightly wrapped up "pappoose," the infant not in the least showing any signs of the effect of having lain uncovered fifteen minutes or longer on a cold, bloody and hard floor, while the mercury registered 28 degrees F.

At 12 m., same day, I was called, on account of violent after-pains, which, however, readily yielded to opiates. On the second day, temperature being up to 100, I put her on quinine and mur. tr. of iron—2 grains and 5 drops respectively—every four hours, and antiseptic vaginal injections twice daily. Temperature did not rise above 100.5, and on the fourth day I detected my patient, in company with several other orientals, men and women, conjointly smoking tobacco, and no doubt enjoying same, out of a "narghile," or Turkish pipe. I discharged the case on the fifth day, and on the eighteenth day I met her on the street, in seemingly good health.

